# Quantifying the impact of a large‐scale opioid agonist treatment program on suicide prevention in New South Wales, Australia: A data‐modeling study

**DOI:** 10.1111/add.70018

**Published:** 2025-02-25

**Authors:** Thomas James Santo, Antoine Chaillon, Natasha Martin, Matthew Hickman, Nicola Jones, Michael Farrell, Chrianna Bharat, Louisa Degenhardt, Annick Borquez

**Affiliations:** ^1^ National Drug and Alcohol Research Centre, Faculty of Medicine & Health University of New South Wales Sydney Australia; ^2^ Division of Infectious Diseases and Global Public Health, School of Medicine University of California San Diego San Diego CA USA; ^3^ Population Health Sciences, Bristol Medical School University of Bristol Bristol UK

**Keywords:** harm reduction, mathematical modeling, opioid agonist treatment, opioid use disorder, public health, suicide

## Abstract

**Aims:**

This study aimed to quantify the population‐level impact of a large‐scale opioid agonist treatment (OAT) program on suicide‐related mortality among people with opioid use disorder (OUD) in New South Wales (NSW), Australia.

**Design:**

This is the first study to use dynamic mathematical modeling to explore the population‐level impact of OAT on suicide mortality. The study used a two‐part approach. First, we analyzed cohort data (2001–2017) to calculate incidence rate ratios (IRRs) and other model parameters related to OAT and suicide risk. Second, findings were applied to model outputs to estimate suicides averted by the NSW OAT program (2001–2020).

**Setting and participants:**

A cohort of 46 845 individuals who received OAT between 2001 and 2017 in community and prison settings in New South Wales, Australia.

**Measurements:**

IRRs for suicide and other model parameters were calculated for individuals on versus off OAT in community and prison settings (2001–2017). These estimates, along with model outputs, were used to determine the number and proportion of suicides averted by the OAT program (2001–2020).

**Findings:**

Receiving OAT was associated with an IRR for suicide of 0.32 [95% confidence interval (CI) = 0.25–0.40] in the community and 0.34 (95% CI = 0.10–1.10) in prison for cohort data analyses (2001–2017). Between 2001 and 2020, the OAT program in NSW averted an estimated 338 suicides [95% credible interval (CrI) = 213–492), with 325 (95% CrI = 202–476) averted in the community and 13 (95% CrI = 0–46) in prison, corresponding to a 35% (95% CrI = 27%–43%) reduction in suicides among those accessing OAT.

**Conclusions:**

The opioid agonist treatment program in New South Wales, Australia, was associated with a 35% reduction in suicide mortality among individuals with opioid use disorder receiving treatment between 2001 and 2020, providing novel evidence of its population‐level impact on suicide prevention.

## INTRODUCTION

Suicide‐related mortality is a critical global public health concern. More than 700 000 people die from suicide each year, which equates to one death from suicide every 40 seconds [1]. Suicide is the 17th leading cause of death worldwide and the fourth leading cause of death among individuals age 15 to 29 years old [1]. Beyond the foremost human cost, suicide is a preventable cause of death that can have substantial psychological and economic effects [2, 3] on families and communities who experience such loss [4].

The urgency to address suicide and related harms is underscored by the United Nations (UN) Sustainable Development Goals [5] and the World Health Organization (WHO) Global Mental Health Action Plan, which both target a one‐third reduction in the global suicide rate by 2030. To achieve these global targets and continue to reduce the burden of suicide globally, it is critical to identify and implement suicide prevention strategies on a broad scale.

Recent evidence in the field of suicide prevention indicates a shift from the traditional focus on individual‐level risk factors and interventions to wider approaches targeting suicide prevention at the population level [6]. In contrast with individual‐level interventions that focus on treatments that reduce suicide ideation, behavior or attempts among individuals at the highest‐risk, population‐level interventions aim to prevent suicide by addressing underlying risk factors and enhancing protective factors to prevent suicide across the wider community or subgroups at high risk [7, 8]. Examples of population‐level interventions include restricting the general public's access to lethal means [8] and reducing social isolation among subgroups at high risk, such as the elderly [9].

Globally, there are an estimated 40.5 million people with opioid use disorder (OUD), a population that is estimated to be at almost eight times the risk of suicide‐related death compared to the general population [10, 11]. Estimates for attempted suicide prevalence among people with OUD range from 20% to 50% [12], and one in 12 deaths among this population are because of suicide [10]. Misclassification of deaths often obscures the true extent of suicide among individuals with OUD, making it difficult to distinguish between overdose and suicide [13]. Shared risk behaviors further complicate the distinction between intentional and accidental overdoses. Despite these misclassifications, overdose and suicide are distinct issues that require different clinical management [14]. Many existing strategies focus on preventing overdose, but fail to address the specific suicide risk faced by people with OUD, making these approaches inadequate [13]. Furthermore, current suicide prevention strategies are generally designed for the broader population and do not adequately meet the needs of individuals with OUD. Therefore, there is an urgent need to develop and implement suicide prevention strategies that are specifically tailored to individuals with OUD [13].

Opioid agonist treatment (OAT), such as methadone and buprenorphine, is an effective treatment for OUD, reducing the risk of multiple causes of death, including suicide [11]. A 2021 meta‐analysis of 14 studies and more than 175 000 people with OUD found that OAT reduces the risk of suicide‐related mortality by more than half [15]. Additionally, more recent studies from Australia and the United Kingdom found that extended periods on OAT are associated with significantly lower risks of self‐harm and suicide at the individual level [16, 17]. However, the population‐level impact of OAT on suicide rates, like many other suicide prevention strategies [6], remains uncertain.

In Australia, suicide was identified as the second leading cause of premature mortality because of injury or disease in 2022, with over 60 000 Australians having died by suicide from 2000 to 2022 including more than 17 000 in New South Wales (NSW) [18, 19]. The OAT program in NSW was established in 1985 and has since achieved high coverage, now reaching approximately 89% of individuals with OUD, according to recent estimates of OUD prevalence [20]. The robust data linkage systems in NSW provide a unique opportunity to estimate the population‐level effects of OAT on suicide by using linked data from the more than 45 000 individuals who have received OAT in NSW to a range of comprehensive state‐wide and federal data on receipt of various health and social services, incarcerations and mortality.

In 2021, Chaillon *et al*. [21] developed a dynamic deterministic compartmental model to assess the impact of the NSW OAT program on overdose and other‐cause mortality from 2001 to 2020. The model accounted for different phases of OAT engagement, including initiation, discontinuation and ongoing treatment and stratified the population by incarceration status to accurately reflect variations in mortality risk, particularly the heightened risk of overdose following OAT cessation and release from incarceration. Chaillon *et al*.’s [21] model estimated that the NSW OAT program averted over 4000 deaths between 2001 and 2020, preventing 53% of overdose deaths and 27% of deaths from other causes.

In this study, we built on the work of Chaillon *et al*. [21] to estimate the impact of OAT on suicide‐related deaths among people who ever received OAT in NSW using a two‐part analysis. First, we updated analyses of the relationship between OAT and suicide risk using NSW administrative data from 2001 to 2017, generating revised estimates of suicides, person‐years and incidence rate ratios (IRRs), extending beyond previous studies that did not conduct community‐specific analyses and were limited to data up to 2012 [22, 23]. We then applied these estimates to the model outputs to specifically estimate the number of suicides potentially averted by the OAT program from 2001 to 2020. By incorporating the specific suicide‐related data, we quantified the contribution of the OAT program to suicide prevention, offering novel insights into its population‐level impact on suicide mortality in NSW.

## METHODS

### Study design, setting and participants

We used data from the Opioid Agonist Treatment Safety (OATS) Study [24]. The reporting in this study is in line with the reporting of studies conducted using observational routinely collected data (RECORD) guidelines [25]. The retrospective cohort comprised all people initiating or maintained on OAT between 2001 and 2017 in NSW, Australia, as recorded in the Controlled Drugs Data Collection (CoDDaC) [20]. Participants were categorized as ‘on OAT’ during periods when they had an active prescription recorded in the CoDDaC, which tracks OAT episodes for individuals receiving methadone or buprenorphine. In NSW, prescribers are required to obtain an authority from the Ministry of Health for each patient to prescribe OAT, valid for a specific period and must notify the system when treatment is discontinued, because an active prescription reflects that this authority has been granted and is still in effect. Prescribers are required by law to inform the Ministry of Health when patients cease treatment [24]. Person‐time was allocated to ‘on OAT’ when individuals were actively in treatment and to ‘off OAT’ when they were not in treatment.

### Data linkage

Four databases were linked to individuals in the CoDDaC by the Centre for Health Record Linkage using probabilistic linkage methods. Records were matched on an individual's name, sex, date of birth and state of residence. The Admitted Patients Data Collection database includes all hospitalizations in NSW. Primary and secondary diagnoses are recorded using the International Statistical Classification of Diseases and Related Health Problems 10th edition (modified for use in Australia; ICD‐10‐AM). Data on state‐wide mental health treatment and incarceration were sourced from the Mental Health and Ambulatory Data Collection and the Re‐offending database, respectively. Mortality and cause of death data for all causes was drawn from the National Death Index (NDI). Further details on the data sources are available elsewhere [24].

### Variables

#### Exposure

OAT exposure time commenced from 1 August 2001 or from first entry into OAT. Participants exited the study on 31 December 2017 or at the time of death. A previous study of the same cohort found that 90% of return treatment episodes occurred within 50 months of the last treatment cessation date [25]. Treatment data was not captured outside of NSW, and any deaths occurring in another state were captured in the NDI.

#### Data analysis for parameter estimation

The cohort has been outlined elsewhere [26]. A flowchart documenting participants excluded at each stage of the data cleaning process is in the Supporting information (Figure S1). The analysis was not pre‐registered and the results should, therefore, be considered exploratory.

The number of suicides and person‐years in the OAT cohort—on and off OAT, in prison and in the community between 2001 and 2017, were extracted. Suicide cases were identified using specific ICD‐10 codes X60–X84, Y87.0 and were categorized within the non‐overdose category, defined by excluding overdose‐related causes (ICD‐10 codes: T400‐T406, T424, T426, T427 and T436). Participants were categorized as being ‘on OAT’ during periods where an active OAT authority was recorded, and ‘off OAT’ during gaps in their treatment. Person‐time was calculated separately for periods on and off OAT. Subsequently, we calculated the IRR and CI of suicide for individuals on versus off OAT and separately for those in prison and in the community. This was done by dividing the number of suicides by the total person‐time for each group (on and off OAT) to determine their respective incidence rates, and then applying the standard IRR and CI formulas [27]. For complete details of the data and calculations, including person‐time and suicide counts, please refer to Table S1 in the Supporting information.

The IRRs from 2001 to 2017 provide the most current estimates of suicide risk in NSW among people with OUD, building on and extending beyond previous studies like Degenhardt *et al*. [23], which presents estimates for all people who received OAT in NSW from 1985 to 2006 (no community‐specific analyses), and Larney *et al*. [22], which provides estimates for prison up until 2012. The original model spans from 2001 to 2020, capturing significant changes in the opioid landscape including the early 2000s heroin shortage and the expansion of OAT with buprenorphine introduced in 2000 and buprenorphine‐naloxone in 2005. Mortality rates among the cohort increased over those two decades, and the IRR estimates used in the model should reflect these changes. By using separate IRRs for prison and community settings, the model accounts for differences in the medications commonly used—such as buprenorphine being more prevalent in prisons compared to methadone's greater use in the community—providing a more accurate reflection of the distinct suicide risks and treatment outcomes in these populations.

Other key parameters required to incorporate the output from our dynamic mathematical model, which projects both overdose and non‐overdose deaths and involves calculating the proportion of non‐overdose deaths attributable to suicide. The model categorizes suicides within the non‐overdose category, defined by excluding overdose‐related causes (ICD‐10 codes: T400–T406, T424, T426, T427 and T436). Using OATS cohort data (2001–2017), we calculated the proportion of suicides among non‐overdose deaths for those on and off OAT in both prison and community settings, and these proportions were applied to the model's non‐overdose death projections to estimate suicide‐specific mortality.

#### Analyzing model outputs with updated cohort data

In our previous modeling study, we used OATS data from 2001 to 2017 to parameterize and calibrate a dynamic deterministic compartmental model of overdose and other‐cause mortality among people with OUD in NSW who ever received OAT between 2001 and 2020. Full detail is provided elsewhere [21], but briefly, the model accounted for different stages of OAT engagement, including initiation, ongoing treatment and discontinuation and stratified the population by incarceration status to reflect variations in mortality risk, particularly the heightened risk of overdose following OAT cessation and release from incarceration. New cohort members entered the model as they initiated OAT treatment for the first time and then progressed through different OAT and incarceration states over time to reproduce observed patterns. They left the model through overdose and other cause death. The model was calibrated using an approximate Bayesian computation sequential Monte Carlo method implemented in R, which incorporated uncertainty in key parameters such as mortality rates, OAT retention and incarceration patterns [28]. The model was calibrated to the annual total number of people in the cohort, the annual number of people on OAT in the community and in prison, the annual proportion currently incarcerated or recently incarcerated and the total number of deaths and the proportion of overdose‐related deaths. The current analysis generates new estimates from our previously developed model to investigate the potential impact of the OAT program on suicide prevention in prison and community settings. Using our original outputs and applying new cohort estimates of the proportion of other‐cause deaths that are because of suicide and of the IRR of suicide among those who are on OAT, disaggregated by incarceration status, we estimated the number of suicides between 2001 and 2020 among those who had accessed OAT, and compared results across two different scenarios:

- Baseline (status quo) scenario with OAT: where we simulated the existing OAT program in NSW and extracted the number of other‐cause deaths occurring over the 2001 to 2020 period among four groups: individuals on OAT in prison, off OAT in prison, on OAT in the community and off OAT in the community. We multiplied these deaths by the percentage of other‐cause deaths attributable to suicide (calculated from the OATS data as described in the previous section among each of those groups) to obtain the number of suicides among each group over 2001 to 2020.
- No OAT program: where we multiplied the inverse of the IRR of suicide associated with OAT in the community and in prison by the number of suicides that occurred among people on OAT in each setting, respectively. The number of suicides among individuals off OAT remained the same across both scenarios because these individuals did not benefit from the protective effect of OAT despite the OAT program being available.

We then calculated the number of suicides averted by the OAT program by subtracting the total number of suicides in the baseline (OAT program) scenario from the total number of suicides in the no OAT scenario. This calculation was also performed separately for the prison and community settings. In addition to the absolute number of suicides averted overall, in the community and in prison, the corresponding proportion of suicides averted overall and by setting was also calculated.

Additionally, we used median ages at death for those who died of suicide and those who died of other causes to calculate the difference and multiplied it by the number of averted suicides to determine total life years gained.

#### Model validation

For validation purposes, we compared the number of suicides with 95% credible intervals (CrIs) estimated by the model in the baseline scenario between 2001 and 2017 (i.e. with the OAT program) versus the observed number of suicides among the OAT cohort in NSW over the same period. We conducted these comparisons for the overall, prison and community settings.

## RESULTS

### Cohort description

There were 46 845 individuals (67.7% male; median age, 32 [IQR = 26–39] years) prescribed OAT in NSW over the study period (Table 1). An average of 20 930 (range across years = 16 405–23 197) received OAT at any one time and 312 756 observed person‐years on OAT. Between 2001 and 2017, there were 433 suicides among the cohort, of which 420 occurred in the community and 13 in prison.

**TABLE 1:** Modeling estimates for the number of suicides in scenarios with and without opioid agonist treatment in NSW from 2001 to 2020.

Baseline scenario (with OAT)	Estimated suicides [credible confidence intervals (95%CrIs)]
**Total suicides in prison**	**27 (95% CrI 19–36)**
Suicides in prison among those on OAT	5 (95% CrI 3–8)
Suicides in prison among those off OAT	22 (95% CrI 16–30)
**Total suicides in community**	**599 (95% CrI 458–750)**
Suicides in community among those on OAT	153 (95% CrI 115–192)
Suicides in community among those off OAT	446 (95% CrI 338–567)
**Total suicides**	**626 (95% CrI 480–782)**

No OAT scenario	
**Total suicides in prison**	**39 (95% CrI 22–74)**
Suicides in prison among those on OAT	—
Suicides in prison among those off OAT	39 (95% CrI 22–74)
**Total suicides in community**	**924 (95% CrI 690–1176)**
Suicides in community among those on OAT	—
Suicides in community among those off OAT	924 (95% CrI 690–1176)
**Total suicides**	**963 (95% CrI 720–1229)**

Suicides averted	
**Total suicides averted (prison and community)**	**338 (95% CrI 213–492)**
Suicides averted in prison	13 (95% CrI −0.5–43)
Suicides averted in community	325 (95% CrI 202–476)
**% suicide averted (prison and community)**	**35% (95% CrI 27%–43%)**
% suicide averted in prison	27% (95% CrI −2%–62%)
% suicide averted in community	35% (95% CrI 27%–43%)

*Note*: Bold indicates that the number is the aggregate number of suicides for those on and off OAT for the specified scenario or setting.

Abbreviations: CrI, credible intervals; NSW, New South Wales; OAT, opioid agonist treatment.

### Re‐analysis of cohort data from 2001 to 2017

In community settings, the IRR of suicide while on OAT was 0.32 (95% CI = 0.25–0.40). In prison settings, the IRR was 0.34 (95% CI = 0.10–1.10). In the community, 10% of non‐overdose deaths on OAT were attributed to suicide with 13% off OAT. In prison, these figures were 21% on OAT and 36% off OAT. These results were used as parameters for the modeling estimations.

### Population suicide estimates: Analysis of model outputs with updated cohort data

**FIGURE 1:**
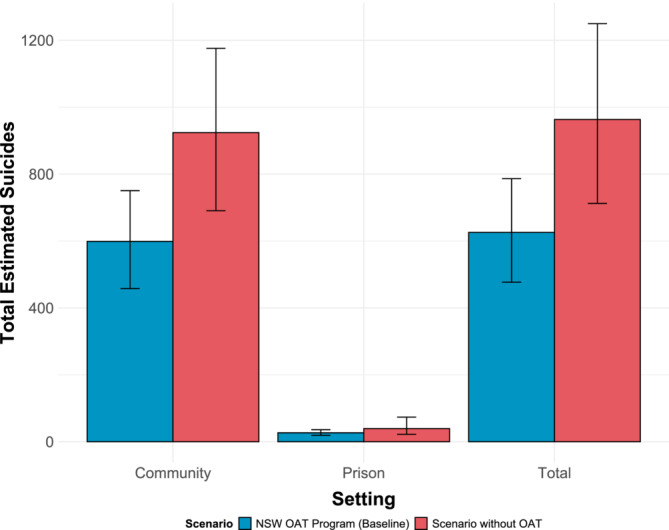
Modeling estimates for the number of suicides among people who accessed opioid agonist treatment (OAT) in New South Wales (NSW) from 2001 to 2020 under the baseline scenario with OAT (blue) and a scenario with no OAT (red). Bars represent modeling estimates for the number of suicides in scenarios with (blue) and no OAT (red) by setting; whiskers represent 95% credible confidence intervals.

Between 2001 and 2020, among individuals with a history of OAT in NSW, the model estimated that 153 (95% CrI = 115–192) suicides occurred among individuals on OAT, whereas 446 (95% CrI = 338–567) suicides occurred among those not receiving OAT. In prison settings, the estimated number of suicides among those on OAT was five (95% CrI = 3–8), compared to 22 (95% CrI = 16–30) among those not on OAT. In our counterfactual scenario, without OAT, the model projected 39 (95% CrI = 22–74) suicides in prison and 924 (95% CrI = 690–1176) in the community, totaling 963 (95% CrI = 720–1229) suicides. Therefore, the OAT program was estimated to have averted 338 (95% CrI = 213–492) suicides between 2001 and 2020, of which 325 (95% CrI = 202–476) and 13 (95% CrI = 0–46) were averted in the community and in prison, respectively (Figure 1). This corresponds to 35% (95% CrI = 27%–43%) of suicide deaths averted in the community and 27% (95% CrI = −2% to 66%) in prison within the cohort of people exposed to OAT (Figure S2).

Based on the median ages at death, for those who died of suicide (39 years, IQR = 32–47) and those who died of other causes (47 years, IQR = 38–54), the 338 averted suicides led to an estimated gain of 2704 life years through the OAT program.

### Model validation

For validation, our model's estimates of suicides under the current program were compared to actual data up to 2017. The model estimated that 22 suicides (95% CrI = 13–25) occurred in prison through 2017, which overestimated the actual number of 13. In the community, the model's estimate of 466 suicides (95% CrI = 357–580) was modestly higher than the actual count of 420. The total suicide estimate by the model was 488 (95% CrI = 375–608), compared to the observed total of 433 which was within the model's CrIs.

## DISCUSSION

### Main findings

Our findings suggest that the OAT program in NSW, Australia, was associated with a reduction in suicide‐related mortality among individuals receiving treatment. Between 2001 and 2020, the program is estimated to have contributed to the prevention of 338 suicides, representing a 35% reduction in suicide deaths within this population. Specifically, our analysis indicates that these interventions were linked to a prevention of 2% of all suicides of the 15 388 that occurred in NSW during the same period [29]. Although its overall impact is modest, the data suggest that the NSW OAT program will contribute toward achieving the WHO and UN Sustainable Development Goal targets to reduce suicide rates by one‐third by 2030 [1, 5]. Additionally, it was estimated that the program helped save approximately 2704 life years over the two‐decade span.

It is likely that averting suicide in people with OUD would reduce health inequalities in NSW as individuals experiencing homelessness, those from lower socio‐economic backgrounds and Aboriginal communities, are disproportionately represented among people with OUD [24] and face a higher risk of suicide [23, 30]. The identification of a population‐level intervention that may have a substantial impact on suicide among these communities is particularly important, given the structural or systemic barriers to mental health and suicide prevention services experienced by marginalized populations in NSW [31] and globally [31, 32]. Ultimately, our study provides foundational evidence for OUD‐specific suicide prevention strategies and demonstrates the potential importance of suicide prevention strategies that are tailored to meet the needs of subpopulations at high risk on a wider‐scale.

The NSW OAT program was estimated to have prevented 13 suicides in prisons, contributing to the overall reduction of suicide deaths in NSW [33]. However, these results should be interpreted with caution because of limitations related to statistical power. Nonetheless, these findings underscore the importance of continuing and possibly expanding OAT programs within prison settings as a potentially effective measure to reduce suicide rates and other causes of mortality among people with OUD.

### Limitations

We acknowledge several limitations. The current study only estimated the number of suicides among individuals exposed to OAT and not the entire population with OUD in NSW. Therefore, our estimates may miss some suicide deaths in people who never enter OAT. However, it is estimated that in NSW nearly 90% of people with OUD are exposed to OAT [20], which would not affect the number of averted deaths, but lead to a slight overestimation of the proportion of averted suicide deaths. Conversely, our findings may have underestimated the proportion of averted suicide deaths because the Chaillon *et al*. [21] model did not account for cohort attrition, specifically the potential for individuals to discontinue OAT and subsequently no longer meet the criteria for OUD.

Another limitation is that our results may have overestimated the reduction in suicide deaths attributed to the NSW OAT program because of confounding factors. Our analyses did not account for potentially confounding factors such as better economic stability and social support, which are associated with both higher OAT engagement [34, 35, 36] and reduced suicide risk [37, 38]. Our analysis, however, only includes people who have at some point been engaged in OAT, therefore, limiting differences between those who died of suicide in and out of OAT. Retention in OAT may also be correlated with engagement with other healthcare services, such as treatment for mental disorders [39], which independently reduces suicide risk [40]. However, it is important to note that the OAT program may have facilitated engagement with such services, mitigating the role of potential confounding [35].

Furthermore, the study only uses treatment data from NSW. This may restrict the generalizability of the findings to other regions, where OAT coverage and retention rates may differ from the high levels observed in NSW [20], and other factors are different, like the availability of fentanyl, which is low in NSW [41]. Additionally, individuals who moved outside NSW were not captured in the data, but given the low levels of interstate migration in Australia, this is unlikely to significantly impact the study's conclusions [42].

This study uses parameter values from 2001 to 2017 that assume the impact of the OAT on suicide is constant over time, which may not accurately reflect variations across different periods. This is particularly important regarding variations in access to different types of OAT medications and changes in barriers to access over this period [43]. Our study also did not differentiate the relative risks of OAT on suicide among those recently and not recently incarcerated because of limited statistical power. We used a single relative risk value for the entire community, which does not adequately reflect the potentially higher suicide risk among individuals recently released from incarceration [33].

Finally, there was greater uncertainty in model projections for the prison setting as there were a low number of events in the cohort informing the model parameters.

### Implications

Our findings suggest that OAT provision may reduce suicide at a population level and provide further support for improving access to OAT globally. Despite strong evidence supporting the effectiveness of OAT in reducing multiple causes of morbidity and mortality among people with OUD, including suicide, the global coverage of OAT remains low [44]. Furthermore, an estimated 90% of people who inject drugs reside in countries where OAT coverage falls below the WHO minimum recommended level of 20%, and most countries are failing to meet the high or even moderate coverage target set out by WHO, UN Office of Drugs and Crime and Joint United Nations Programme on HIV/AIDS [44]. Our findings highlight the urgent need for increased access to OAT programs worldwide, particularly given that expanding these programs has the potential to reduce suicide‐related deaths among individuals with OUD by more than one‐third, as indicated by our findings.

Future research should examine the effects of expanding OAT programs on suicide rates in specific populations and settings, as well as the effect of OAT in combination with other suicide prevention strategies, such as access to mental health services for comorbid mental disorders [16, 45], routine screening for suicide ideation among people with OUD [46] and emergency department follow‐up of individuals with OUD who present with self‐harm [16, 47]. Expanding access to suicide prevention services in conjunction with OAT is underscored by findings from previous analyses of the cohort of people exposed to OAT in NSW, which found that 66% of suicides involved violent methods such as hanging, 24% were because of non‐opioid overdoses and less than 10% involved opioid overdose [16]. This emphasizes the importance of incorporating broader, more comprehensive suicide prevention approaches within OAT services, because focusing only on opioid overdose prevention may miss the majority of suicides [14, 16].

In summary, our findings underscore the critical role of OAT as a public health intervention in mitigating suicide risk among people with OUD, supporting its expansion and integration into broader public health strategies that aim to reduce population‐level suicide rates.

## AUTHOR CONTRIBUTIONS

**Thomas James Santo Jr:** Data curation (equal); formal analysis (equal); investigation (equal); methodology (equal); visualization (equal); writing—original draft (equal); writing—review and editing (equal). **Antoine Chaillon:** Conceptualization (equal); data curation (equal); formal analysis (equal); investigation (equal); writing—original draft (equal); writing—review and editing (equal). **Natasha Martin:** Conceptualization (equal); formal analysis (equal); investigation (equal); methodology (equal); supervision (equal); writing—review and editing (equal). **Matthew Hickman:** Conceptualization (equal); investigation (equal); supervision (equal); writing—review and editing (equal). **Nicola Jones:** Data curation (equal); formal analysis (equal); writing—review and editing (equal). **Michael Farrell:** Funding acquisition (equal); investigation (equal); resources (equal); supervision (equal); writing—review and editing (equal). **Chrianna Bharat:** Data curation (equal); formal analysis (equal); investigation (equal); writing—review and editing (equal). **Louisa Degenhardt:** Conceptualization (equal); funding acquisition (equal); investigation (equal); resources (equal); supervision (equal); writing—review and editing (equal). **Annick Borquez:** Conceptualization (equal); formal analysis (equal); investigation (equal); methodology (equal); supervision (equal); writing—original draft (equal); writing—review and editing (equal).

## DECLARATION OF INTERESTS

In the past 3 years, L.D. and M.F. have received investigator‐initiated untied educational grants for studies of opioid medications in Australia from Indivior and Seqirus. All other authors have no conflicts of interest to declare.

## Supporting information


**TABLE S1.** Definitions for suicide related deaths and person years on and off oat and in prison and the community using administrative data from.
**FIGURE S1.** Flowchart of participant inclusion.
**FIGURE S2.** Modeling estimates for the percentage of suicides averted among people who have received oat in scenarios with and without opioid agonist treatment in NSW from 2001 to 2020.

## Data Availability

The data are not available due to privacy or ethical restrictions.
