# Supplementary material for: Quantifying the impact of a large‐scale opioid agonist treatment program on suicide prevention in New South Wales, Australia: A data‐modeling study
Source: Addiction. 2025 Feb 25;120(8):1601–9. doi: 10.1111/add.70018 (PMC12215286; doi:10.1111/add.70018)
Supplement: Supplementary file 1 — TABLE S1. Definitions for suicide related deaths and person years on and off oat and in prison and the community using administrative data from. FIGURE S1. Flowchart of participant inclusion. FIGURE S2. Modeling estimates for the percentage of suicides averted among people who have received oat in scenarios with and without opioid agonist treatment in NSW from 2001 to 2020. [file ADD-120-1601-s001.docx]

# Supplementary Material: Modeling the population-level impact of opioid agonist treatment on suicide among people accessing treatment between 2001 and 2020 in New South Wales, Australia

## eTable 1. Definitions for suicide related deaths and person years on and off OAT and in prison and the community using administrative data from

| **Measure** | **Definition** | **Total (2001-2017)** |
| --- | --- | --- |
| Person years in OAT | Based on active authority records | 282850 |
| Person years out of OAT | For people who have an active record between 2001 and 2017 and are not on treatment but have been on OAT previously | 251861 |
| Suicide ICD 10 codes | "X60", "X61", "X62", "X63", "X64", "X65", "X66", "X67", "X68", "X69", "X70", "X71", "X72", "X73", "X74", "X75", "X76", "X77", "X78", "X79", "X80", "X81", "X82", "X83", "X84", "Y87.0" | 433 |
| Person years incarcerated and in OAT | People are recorded as being on OAT and incarcerated if they have a custody record and an active OAT authority | 20158 |
| Person years incarcerated and off OAT | Based on custody records and no active OAT authority | 15376 |
| Number of suicide incarcerated and on OAT | Number of suicides On OAT and incarcerated 2001-2017 | 4 |
| Count suicide incarcerated and off OAT | Number of suicides Off OAT and incarcerated 2001-2017 | 9 |
| Person years in the community on OAT | On OAT and not incarcerated | 262692 |
| Person years in the community off OAT | Off OAT and not incarcerated | 236485 |
| Count suicide in the community on OAT | Number of suicides On OAT and not incarcerated 2001-2017 | 110 |
| Count suicide in the community off OAT | Number of suicides Off OAT and not incarcerated 2001-2017 | 310 |

**Notes on rules applied to extract death data:**

- Death records come from the National Death Index.
- OAT while incarcerated is derived from incarceration records and an active OAT authority.
- This extract has not applied the 6-day rule for consecutive OAT episode being considered one episode if an episode starts within 6 days of a prior OAT episode ceasing which has typically been used in previous analysis.
- This extract has not applied any cut-off time for post OAT (i.e. someone who last had OAT in 2001 and did not die during the follow-up period will have 16 years of out of OAT person-time), we do not have visibility of people leaving the State.
- This extract has applied a one day rule for deaths, a death occurring one day after an OAT episode ceased, i.e. deaths that occur a day after OAT was cancelled, has been considered an in treatment event.
- Additionally, if a participant died in hospital and was on OAT prior to hospitalisation it is considered an in treatment event.
- This extract has had participants removed if identified as poor linkage matches at data cleaning stage (i.e. people with multiple year and month of birth and events after death

## eFigure 1. Flowchart of participant inclusion


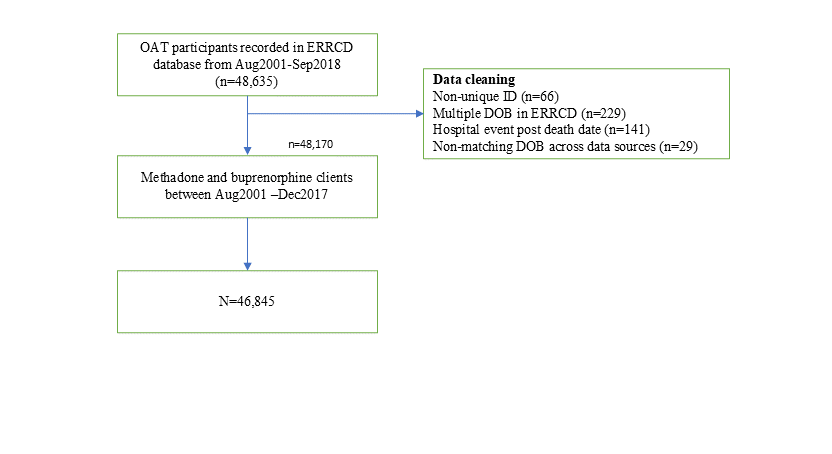


**Table Notes:** Methadone and buprenorphine (including buprenorphine-naloxone) are available in public, private, and carceral settings in NSW for the management of OUD. Prescribers are required to submit an “Authority to Prescribe” form for people entering treatment and are required to inform the regulatory body when dosage or dispense setting changes and if people exit treatment. Medication changes between methadone and buprenorphine are also recorded.

## eFigure 2: Modeling estimates for the percentage of suicides averted among people who have received OAT in scenarios with and without Opioid Agonist Treatment in NSW from 2001-2020


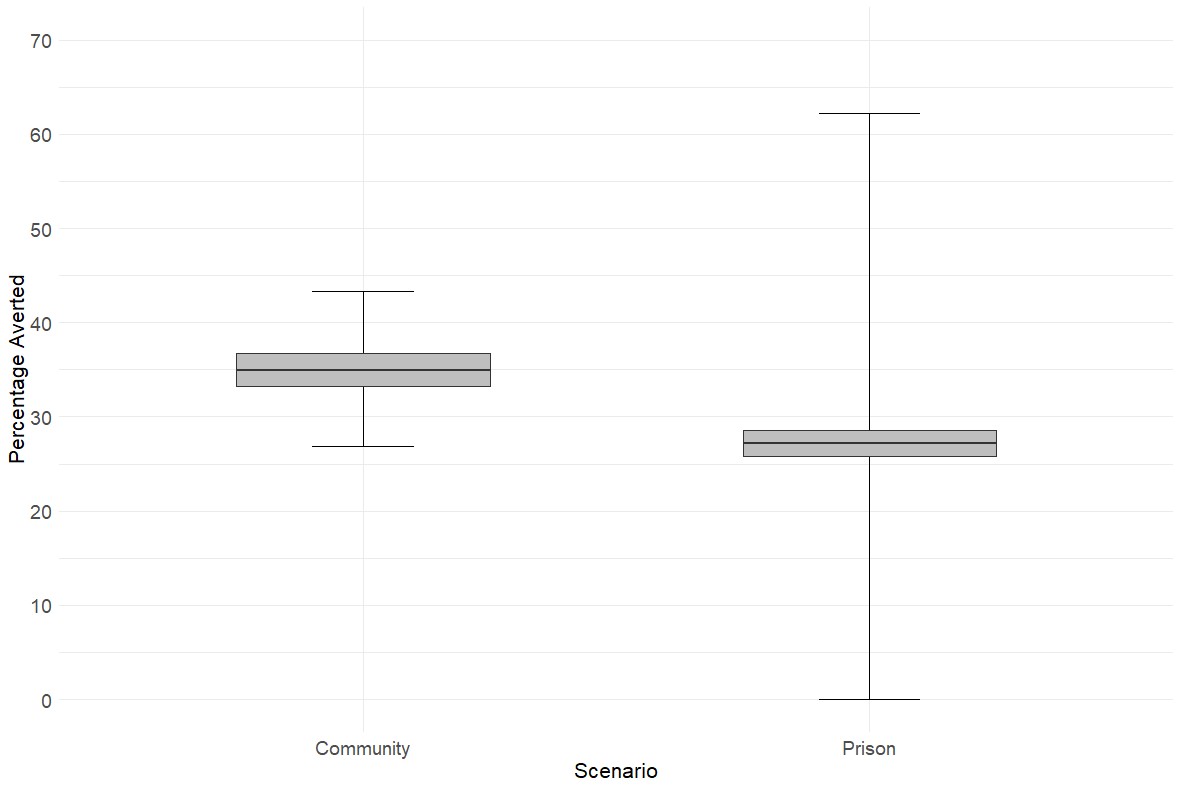

**Notes:** Boxes represent modeling estimates for the percentage of suicides in scenarios with and without OAT by setting; Whiskers represent 95% Credible Confidence Intervals
